# Microfluidic Devices for Drug Assays

**DOI:** 10.3390/ht7020018

**Published:** 2018-06-20

**Authors:** Clément Regnault, Dharmendra S. Dheeman, Axel Hochstetter

**Affiliations:** 1 Wellcome Centre for Molecular Parasitology, Institute of Infection, Immunity and Inflammation, College of Medical, Veterinary and Life Sciences, University of Glasgow, Glasgow G12 8TA, UK; c.regnault.1@research.gla.ac.uk (C.R.); dheeman@gmail.com (D.S.D.); 2 Division of Biomedical Engineering, School of Engineering, University of Glasgow, Glasgow G12 8LT, UK

**Keywords:** microfluidics, drug assays, high-throughput, review, well plates, droplet-microfluidics, drug discovery, continuous flow microfluidics

## Abstract

In this review, we give an overview of the current state of microfluidic-based high-throughput drug assays. In this highly interdisciplinary research field, various approaches have been applied to high-throughput drug screening, including microtiter plate, droplets microfluidics as well as continuous flow, diffusion and concentration gradients-based microfluidic drug assays. Therefore, we reviewed over 100 recent publications in the field and sorted them according to their *microfluidic* approach. As a result, we are showcasing, comparing and discussing broadly applied approaches as well as singular promising ones that might contribute to shaping the future of this field.

## 1. Introduction

### 1.1. Microfluidics

Microfluidics is a field of science studying fluids (i.e., liquids and gasses) on a microscopic scale. Therefore, fluids are confined in devices that have a significant dimension (e.g., the height or the width) on the micrometer scale. Due to this extreme confinement, the volumes used for drug assays and similar studies are tiny (milliliters to femtoliters), and special physics apply. In short, in a microfluidic device there are only laminar flows and no turbulences, which grants an extremely high control over the fluids employed, diffusion of drugs and the progress of reactions. While we do present a short introduction into the field, this review focuses on microfluidic (high-throughput) drug assays with the most noteworthy developments in the recent years. We would like to point interested reader towards several germane publications that give an in-depth dive into the field of microfluidics [1] and its drug-related applications [2,3,4,5,6,7,8,9,10].

### 1.2. The Physics of Microfluidics

One of the most important parameters describing the flow in microfluidic systems is the Reynold’s number (*Re*), which is defined as the ratio of inertial to viscous forces:

(1) $$Re=\;\frac{\rho vL}{\eta },$$

where ρ is the fluid’s density (kg/m^3^), ν is the mean velocity (m/s), *L* is the characteristic dimension of the channel (e.g., the channels diameter, height or width; m) and η is the dynamic viscosity of the fluid (kg/(m·s)). For *Re* > 1, the propulsion of a body is dominated by its inertia, like a swimmer who stops to actively swim but still is moving forward. For *Re* < 1, the viscous forces dominate the propulsion, like a swimmer in honey: the moment she stops to actively push herself through honey, she is immobile. *Re* also explains different flow regimes such as *laminar* (up to *Re* = 10) and *turbulent* (*Re* > 2000). The numbers given here are empiric for generic microfluidic setups, where in turbulent flows are caused by perturbations. Specific setups can achieve laminar flow regimes at higher Reynolds numbers [11]. For laminar flow systems the streamlines are parallel, and mixing is strictly diffusion governed. For turbulent flow systems the streamlines are chaotic and thus mixing can be rapid (but is hard to control and calculate). In microfluidic devices, *Re* is often below unity resulting in *laminar* flows throughout the device. It is also possible to compartmentalize the fluid into tiny droplets, with each droplet acting as a unique microreactor. Often, two hydrophilic liquids co-flowing in a microfluidic channel (e.g., a cell dispersion and a dye or stain) get combined into a droplet (where chaotic convection results in rapid mixing) by being surrounding by a hydrophobic oil. Adding surfactants stabilizes the droplets and inhibits the coalescing of neighboring droplets. In this way, cells can be stained or lysed directly upon encapsulation, stored safely, and individually analyzed, while no cellular contents are released before compartmentalization.

### 1.3. Manufacturing Microfluidic Devices

Devices for microfluidics have a significant dimension (e.g., height or width) in the micrometer regime. To accurately create these devices, several technologies have been employed, e.g., 3D printing [12,13], etching and micromachining [14] and standard soft-lithography techniques [15,16]. These techniques are carried out in clean-room facilities to avoid artifacts from airborne pollution (e.g., dust). 

While both continuous flow microfluidics and droplet microfluidic approaches [8,17,18] depend on elaborate device designs to carry-out high-throughput drug assays, well plate-based approaches rely instead on high level parallelization and large robotic machinery. In addition, a growing number of companies offer services ranging from fabricating microfluidic devices to conducting drug assays themselves (e.g., 3D Biomatrix, Fluigent, Dolomite, uFluidix, etc.).

### 1.4. Drug Discovery and Drug Assays

Drug discovery in this day and age is a multibillion-dollar venture requiring a significant upfront capital investment with no guarantee of success. Based on the past pharmaceutical industry experience in drug discovery and development, it takes approximately 15 years and about $1.2 to $1.8 billion to develop a small drug molecule [19]. Despite this heavy investment of time and capital, seven out of 10 drugs are likely to fail to recover the research and development (R&D) costs incurred [20]. Therefore, to stay competitive, the pharmaceutical industry is under increasing pressure to discover new drug entities and to bring down the cost of drug development [21]. With the advent of genomics and combinatorial chemistry and the adoption of high-throughput screening (HTS) methodologies [22] there has been a deluge of data on the potential therapeutic drug targets and libraries of drug molecules to be screened. This has necessitated the development of cost effective methodologies to efficiently screen a vast number of compounds against a variety of therapeutic targets.

### 1.5. Definition: High-Throughput Screening (Well Plate vs. Droplet vs. Continuous Flow Microfluidics)

**Figure 1:**
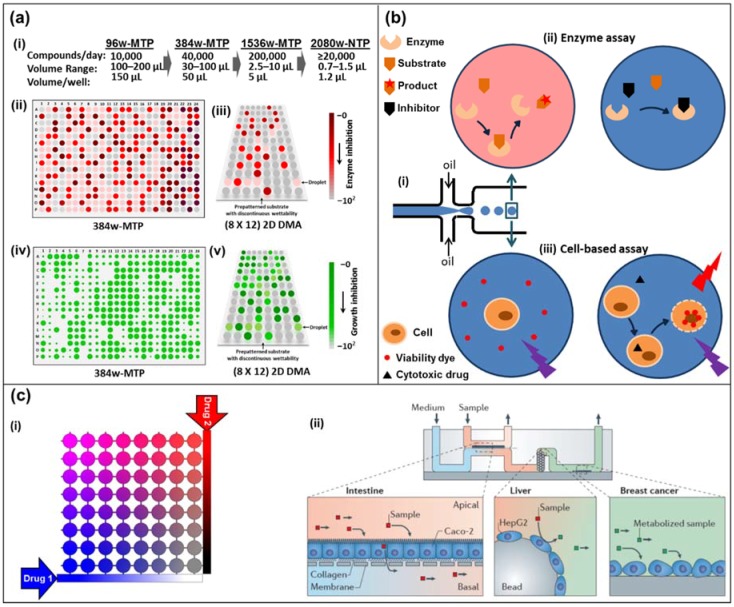
(**a**) Miniaturization of high-throughput screening in array formats. (i) Miniaturization of well-plate screening formats. (ii) Schematics of high-throughput screening using a colorimetric assay in 384-well microtiter plate (384w-MTP) and (iii) in 2D droplet microarrays (2D DMAs) on a planar substrate. The intensity of the red-brown color indicates the extent of the reaction (conversely, lack thereof depicts inhibition). (iv) Schematics of high-throughput screening using a cell-proliferation fluorescent marker on 384w-MTP, and (v) in 2D DMA on a planar substrate. The florescence signal is proportional to the extent of cell viability/growth (conversely, no signal means death). (**b**) Droplet-based microfluidics. (i) Microdroplet formation using the flow-focusing geometry. Droplets are generated at a frequency of 1–1000 droplets/s by shearing an aqueous stream with oil in two directions. (ii) An enzyme and its substrate are encapsulated in a microdroplet (top left). The formation of a colored product can be measured by absorbance. In the presence of an inhibitor in the droplet, product formation is blocked (top right). (iii) A cell and a cell-impermeable viability dye are encapsulated in a microdroplet with (right bottom) or without (left bottom) a cytotoxic drug. Upon cell death, the viability dye binds to the DNA and emits a red fluorescence signal. (**c**) Diffusion-based microfluidics. (i) Schematic of an 8 × 8 microfluidic diffusion array. Initially, cells (or target molecules) are seeded into the individual chambers (circles). Through a system of reservoirs, pumps and channels, two compounds (e.g., drugs, sensitizers) are diffused into the chambers, each chamber having their individual concentration pair of (Drug 1) and (Drug 2). Over the course of the experiment (e.g., 3–5 days) the chambers can be supplied with fresh media and drug solution while metabolite samples can be collected and analyzed. (ii) A microdevice containing interconnected cell culture microchambers was used to develop a multi-organ model that integrated microfluidic culture of intestinal epithelial cells, hepatocytes and breast cancer cells to simulate absorption, metabolism and activity of anticancer drugs. Adapted by permission from Springer Nature: Nature, Nature Reviews Drug Discovery, Organs-on-chips at the frontiers of drug discovery, [25].

There are three different concepts, all of which are referred to as *microfluidic high-throughput drug assays* (see Figure 1):

(a) Traditionally, high-throughput drug assays are carried out in microtiter well plates, where the drug solutions are added to either target cells or target molecules. The entire well plates are incubated for days before all the wells are read out in parallel. The high level of parallelization and the usage of mere microliters of drugs gave rise to the *microfluidic high-throughput* label.
(b) With the emerging field of microfluidics advancing rapidly, drug assays have been developed in two approaches. The first approach is based on droplet microfluidics, where each droplet’s contents correspond to a well from a microtiter plate. Hundreds of these droplets can be generated every second, stored for the incubation time, and read out automatically at similar speeds.
(c) Another approach stems from microfluidic devices operating with a single (aqueous) phase, where drug concentrations are diffusion-controlled, and can be changed periodically and rapidly to obtain individual results within minutes instead of days. This also allows us to test drugs on entire tissues and *organs-on-a-chip* at the lower frequencies.

### 1.6. Cell-Based vs. Molecular Assays

Another aspect helps to categorize the multitude of drug assays that have been published in the recent years: In the most targeted approaches, assays are developed to study the effect of a drug on a molecular process, such as an enzyme activity. These biochemical assays are also important to study the mode of action of drug candidates. However, in the cellular environment, other biophysical or biochemical processes may interfere with the drug, thereby limiting its effect. Therefore, drug assays are also developed to study how entire cells respond to treatment with specific compounds (usually in terms of viability). 

Another important aspect of drug development lies in cytotoxicity studies at the organ level. To address this in a non-invasive way, new approaches have emerged in the field of microfluidic 3D cellular scaffolding and organ-on-a-chip. Drug assays using these technologies (with a relatively low throughput) have already been reported [23,24,25,26,27,28].

## 2. Well Plate Setups

### 2.1. The 96-Well Microtiter Plate as an Industrial Standard

Drug screening is well-established in academia and the pharmaceutical industry. The screening of compounds for activity against specific therapeutic targets has traditionally been done manually with typical throughputs of around a 1000 assay per day [29]. The 96-well microtiter plate (96w-MTP) is currently the standard format of miniaturization and automation for bioassays in drug discovery: Lariosa-Willingham et al. [30] reported a HTS assay in 96w-MTP format to identify new therapeutic agents that accelerate the differentiation of immature oligodendrocyte precursor cells into mature, myelin-producing oligodendrocytes (OLs). Myelin-producing oligodendrocytes promote the remyelination of axons following an immune attack. A total of 727 compounds were tested using an OL differentiation assay based on immunofluorescent image quantification. Bowden et al. [31] explored a drug repurposing library of 725 compounds using a HTS in 96w-MTP format for firefly-luciferase-expression in *Sarcocystis neurona*, the primary causative agent of equine protozoal myeloencephalitis. 

### 2.2. Miniaturization

With the increased availability of enhanced combinatorial compound libraries there has been more demand for increased throughput [32], which has led to the miniaturization of well-plate assay formats [33]. The sample volume of a typical 96w-MTP format assay is ca. 100 μL with a throughput potential of around 10,000 assays per day. A further 3-fold throughput gain has been realized through miniaturization—resulting in the 384-well microtiter plate (384w-MTP) format with assay volumes of ca. 10 μL [29]. Most assays, molecular or cell-based, can be fit to 384w-MTP format without problems, thus it has become the established well plate format for compound storage and screening assays. Recently, Baumann et al. [34] reported a cell free in vitro translation assay, based on activity of click beetle red luciferase reporter gene. This was used to screen novel antimalarial protein translation inhibitors in a 384w-MTP format using a standard spectrofluorometer. Similarly, Brennan and Tillitt [35] adapted a dual enzyme and protein assay to the 384w-MTP format to screen endocrine-active substances with no loss of sensitivity. 

### 2.3. Ultra-High-Throughput Screening and Low Volumes

The increasing push towards higher density assay formats has led to the development of even higher-throughput/lower-volume assay formats, i.e., ultra-high-throughput screening (uHTS) formats. uHTS in 1536-well microtiter plate (1536w-MTP) formats can enable assay volumes of less than 5 μL and a throughput of more than a 100,000 assays per day [36]. Auld et al. [37] screened two low molecular weight compound libraries (44,000 compounds) and a purified natural products library (2583 compounds) to describe the inhibitor profile of purified enzyme, TurboLuc, and also showed the use of this reporter enzyme in cell-based assays employing uHTS in 1536w-MTP. Norcliffe et al. [38] adopted an auxotrophic yeast-based uHTS approach for inhibitors of the *Leishmania* enzyme (inositol phosphorylceramide synthase). A 1.8 million compound library was screened against an auxotrophic *Saccharomyces cerevisiae* mutant and the cell growth was measured by fluorescence.

The ongoing trend to further miniaturize well plate formats resulted in the 2080-well nanotiter plate (2080w-NTP), the 3456-well plate and a low-fluorescence plastic 9600-well plate. These formats enable high-throughput assays at volumes as low as 0.2 μL to 1.0 μL [39]. Even a 20,000-well plate format with a well volume of merely 25 nL has been reported [40], but these formats are not readily commercially available. 

### 2.4. Challenges and (Well-Less) Alternatives

Despite the advantages gained through miniaturization, ultra-high-density plates pose technical challenges. The small reaction volumes in these wells have a high surface-to-volume ratio leading to increased reagent adsorption, evaporation and stability issues. Screening in ultra-high-density plates also faces the problems associated with gaseous exchange of well-plate cell cultures [41,42]. For plate densities higher than 384 wells, effective mixing is a challenge given the potential viscosity and volatility of assay reagents [43]. 

Recently, Hernandez-Perez et al. [44] and Küster et al. [45] demonstrated that—like well plate formats where reactions are confined to isolated wells—HTS reactions can also be isolated in sessile 2D droplet microarrays (DMAs) on planar surfaces. These sessile droplets contain volumes of 0.1 nL to 10 μL, and can potentially increase in the throughput to 6144 (4 × 1536) droplets per plate [46]. Sessile DMAs can be viewed as well-plates without walls and have emerged as an alternative platform offering greater versatility and simplicity of operation [46]. Well-plate liquid handling instrumentation is easily adaptable to sessile 2D DMAs. An entirely motorized, hydrophobic handling stage for sessile droplets has been developed by Kong et al. [47]. Though, sessile 2D DMAs have been exploited in few instances to drive assay reactions and to concentrate analytes, there are still challenges of controlling evaporation of droplets [44]. In a related approach, Casavant et al. suspended a droplet within a microfluidic setup that has neither a roof nor a floor. In this suspended microfluidic setup, they studied cellular growth and its suppression using laminin [48]. A different route to further adapt well-based assays has been developed by Berry et al.: standard Transwell plates are transformed into multi-colony assays by inserting hydrogel walls [49].

## 3. Droplet Microfluidics-Based Assays

Droplet-based microfluidic platforms have also been increasingly applied to high-throughput drug screening. Microdroplets arise from mixing immiscible fluids in a microfluidics channel. A variety of device geometries can be used for droplet generation, as reviewed by Zhu and Wang [50]. This in vitro compartmentalization of biological systems enables to perform biochemical reactions or cell-based assays in volumes ranging from nanoliters down to femtoliters [51]. Reactions and analyses in these microdroplets are facilitated by the fact that they can easily be mixed [52], merged [53], split [54], trapped [55], transported for off-chip incubation [56] or sorted [57]. It has the advantage of being highly controllable and shows great potential for performing high-throughput experiments.

### Droplet Cytotoxicity Assays & Encapsulated Monoclonal Antibody Screening

Brouzes et al. pioneered in this field by developing a platform for cytotoxicity screening. In this approach, a library of drug-encapsulated microdroplets (fluorescently-labelled for downstream identification) was generated. These were subsequently merged one-to-one with cell-containing droplets at high-throughput (100 events/s). The resulting microreactors were incubated for 24 h, before being merged with fluorescent live/dead stain-containing droplets. The final on-chip fluorescent read-out enabled to both identify library members and assess their cytotoxic properties [18]. In an approach similar to Bithi et al. [58], Wong et al. [59] designed an array of 48 droplet-formation wells. This enabled them to generate microdroplets containing cancer cells with a drug and a viability dye. After a 24 h on-chip incubation, fluorescence microscopy images were captured, and cell viability was analyzed using a specific algorithm. The drug potency values obtained using this platform were comparable to the ones measured in the 96- and 384-well plate formats. In particular, the authors tested the cytotoxicity of drugs against human primary nasopharyngeal tumor samples and reported the screening of 5 conditions in parallel on 80,000 cells at the single-cell level. This is of particular importance in the context of rare cells, such as circulating tumor cells that occur in the blood at concentrations as low as 1 cell per mL. A droplet-based microfluidics platform was also developed for assessing drug compounds cytotoxicity in 3D spheroids of cancer cells [60].

**Figure 2:**
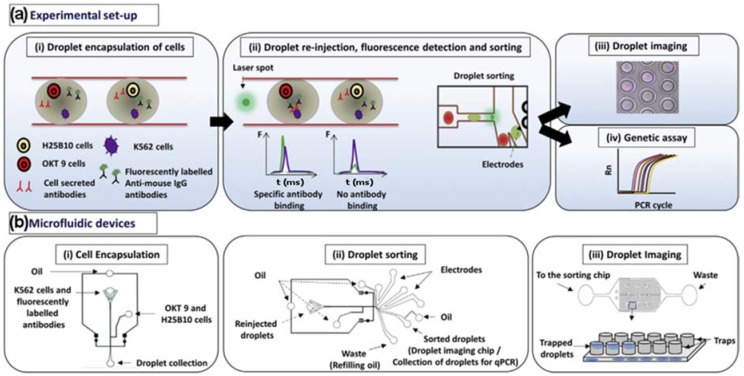
(**a**) (i) K562 target cells were co-encapsulated in droplets with OKT 9 and H25B10 hybridoma cells, along with fluorescently labeled anti-mouse antibodies. K562 cells were stained with violet stain in all experiments, whereas OKT 9 cells were stained with red dye only for imaging experiments. (ii) The droplets were re-injected into a sorting device and excited with laser. Upon specific binding of OKT 9 secreted antibodies to K562 cell surface, a sharp fluorescence peak was observed. However, non-specific antibodies secreted by H25B10 cells failed to show binding to K562 cells, as a result fluorescence peak was not observed. Based on the fluorescence peak data, droplets were sorted by dielectrophoresis mechanism. (iii) The individual droplets were captured in traps and imaged to determine the cell occupancy before and after droplet sorting thereby revealing the sorting efficiency. (iv) Alternatively, the enriched cell population obtained after antibody binding-based droplet sorting, was processed for a real-time polymerase chain reaction (PCR) assay to determine the sorting efficiency. (**b**) (i) The microfluidic device used for generating aqueous droplets in oil has been shown. The K562 cells and fluorescently labeled anti-mouse antibodies were introduced together, whereas OKT 9 and H25B10 cells were introduced through a different inlet, as indicated by arrows. (ii) The microfluidic device used for droplet sorting has been shown. The functions of various inlets have been indicated by arrows. (iii) The microfluidic device used for trapping droplets has been shown. As depicted in the cartoon, inverted traps capture the droplets, which can then be imaged. Adapted from [63] in accordance with the terms of the Creative Commons Attribution License (CC BY).

Monoclonal antibodies are used in the treatment of a vast range of diseases, particularly in cancer therapy [61]. One key aspect of this is the binding of antibodies to cell-surface receptors. Shembekar et al. [62] have developed an approach to screen for the specific binding of antibodies directly to a single target cell (Figure 2). They co-encapsulated a target cell with an antibody-secreting cell and a fluorescently-labelled secondary antibody in a ~100 µm droplet. Specific binding of the secreted antibody to its target cell led to localization of the secondary antibody to the cell surface, thereby generating a strong fluorescence signal that can be easily detected by fluorescence-activated cell sorting (FACS) or fluorescence microscopy. Droplets showing a positive signal were then successfully sorted for enrichment and subjected to downstream imaging and real-time polymerase chain reaction (PCR) analyses. This platform could reach a throughput of 1,000,000 droplets processed per screen, of which 80,000 contained both cell types. This approach offers the potential to screen primary cells for secretion of specific antibodies.

Cell-free drug screening is also being implemented in the microfluidics droplet format. Mao et al. designed a chip to perform enzyme assays [64]. Droplets encapsulating an enzyme, its substrate and a potential inhibitory compound were generated in the first part of the device. They were then directed into a mixing section and a reaction section, which enables to control reaction time. Product formation was monitored by absorbance measurement, thereby assessing the inhibitory potency of the compound. Courtney et al. developed a device to assess the potential of drug compounds as inhibitors of tau-peptide activation, a mechanism reportedly involved in the pathology of Alzheimer’s disease [65]. Importantly, sample consumption was decreased by 200-fold, and the assay time was reduced from 2 h to 2.5 min as compared to the 96-w-MTP format, while comparable results were obtained using orange G as a reference model.

## 4. Continuous Flow, Diffusion and Concentration Gradients-Based Microfluidic Drug Assays 

In recent years, diffusion-based microfluidic drug-assays have also been attempted on the single cellular level. An et al. generated a microfluidic chip with 8 × 8 chambers—each chamber filled with viable prostate cancer cells and distinct concentrations of a sensitizer and a drug [66]. Their microfluidic setup allowed for a diffusion-based drug assay with regular medium exchange over a couple of days. The results for the sensitizer and drug efficacy using the microfluidic approach were comparable to a 96 well-plate experiment they carried out in parallel, but showed more pronounced efficacies, due to the repeated drug exposure, which was not feasible in the well-plate assay [66].

**Figure 3:**
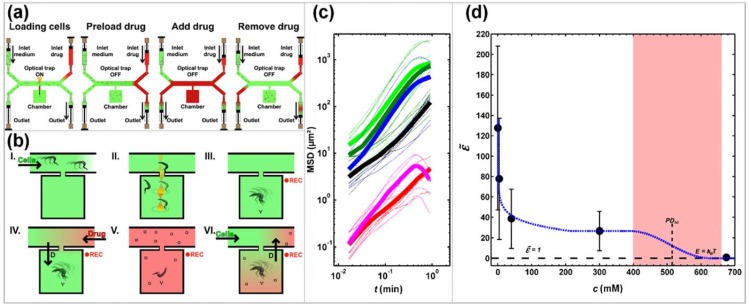
Schematic representation of the experimental design at the level of (**a**) the device and (**b**) the microchamber and analysis of the impact of 2-deoxy-d-glucose (2DG) on trypanosome propulsion (**c**,**d**). (**a**,**b**): The device consists of microchambers along a main channel that connects the cell side with the drug side. Both sides have an inlet and an outlet at the end of the respective Y-split. Panels (I-VI) illustrate individual operational steps in (**a**) and the resulting relative concentrations of solutions in (**b**). (I) Channels and microchambers are filled with culture medium, (II) in the absence of applied flow, individual cells are trapped by optical tweezers and moved into a microchamber. (III) Cell movements in a drug-free confinement are recorded while the drug side is flushed with drug-containing solution (Drug) to remove culture medium. (IV) Drug is pumped through the main channel and drug molecules diffuse into the chambers while cell movements are recorded. (V) At maximum drug concentration, the cell side is flushed with culture medium. (VI) Culture medium is pumped from the inlet on the cell-side through the main channel and recording can be stopped. (**c**) Mean squared displacement (MSD) of trypanosomes in identical chambers but at different concentrations of 2DG in the medium (bright green: drug-free culture medium, dark green: C_2DG_ = 4 mM, blue: C_2DG_ = 40 mM, black: C_2DG_ = 300 mM, magenta: fixed with glutaraldehyde (GA), red: paralyzed; C_2DG_ = 700 mM). Thin lines are MSD of individual cells; bold lines are averages of a number of cells at the same 2DG concentration. (**d**) The motility factor $\tilde{\epsilon }$ plotted against the concentration of 2DG in the medium. The dashed black line represents a motility factor $\tilde{\epsilon }$ = 1, which means that the propulsion energy of a trypanosome equals the thermal energy, *k_B_T*. The area shaded in red corresponds to the concentration range of reversible cell paralysis. Dashed blue line serves as a guide to the eye. Adapted from [67].

Another diffusion-based microfluidic cell viability drug assay was published by Hochstetter et al., who also focused on the single cell level but delivered results much faster [67]. By analyzing the motility of cells in dependence to drug exposure (see Figure 3), they could identify dose-dependent drug effects within seconds. Motile cells (*Trypanosoma brucei brucei*) were singled out into microchambers and their motility was recorded using high-speed video cameras. Then they were exposed to drugs (e.g., suramin, 2-deoxy-d-glucose) in a diffusion-controlled concentration ramping and their movements were still being recorded (see Figure 3a,b). By analyzing the changes in their motility (e.g., their mean squared displacement, see Figure 3c) it was possible to find a range of drug effects from a mild slowing down to reversible paralysis to cell death for concentrations ranging over three orders of magnitude within minutes of experimental time (see Figure 3d) [67]. One important aspect in this setup was the dimensions of the long and thin connecting channels between the main channel and the micro chambers, which ensured a controlled diffusion and inhibited turbulences and cross-flow [68]. Ding et al. used a similar motility assay to analyze drug efficacy in *Caenorhabditis elegans* [69].

### Further Notable Approaches

Wu et al. even developed a 3D-printable platform for experiments with controlled perfusion in microfluidic paper-based analytical devices (µPADs) [24]. Their approach allows for using µPADs for drug screening and organ-on-a-chip setups, thus combining two very promising techniques for future application in efficient HTS. Whitesides and co-workers used exchangeable stacks of paper (impregnated with cells in a hydrogel) to generate a tissue-like scaffolding for three-dimensional cell cultures, which can potentially be employed for drug assays in the future [70]. Generally, microfluidics has been employed to generate various analytical devices, opening up research niches like paper microfluidics [71], digital microfluidics [72,73], microfluidic electrochemical assays [74] and 3D printing microfluidics [13], which have the potential to be applied to drug assays as well. Digital microfluidics has already been employed for combinatorial synthesis of peptidomimetics [75], which could prove very useful not only to conduct drug assays on a chip, but also to generate drug libraries on the same compound chip.

Table 1 comparatively summarizes the main advantages and drawbacks of the three major drug assay technologies described here. 

## 5. Discussion

The traditional well-plate-based drug assays are still widely used in industry, thanks to standardized formats that enable automated handling. Over the last few decades, we have observed a clear trend towards miniaturization in this field, which culminates with the droplets microarray format. The latter allows for performing biochemical reactions in volumes as low as 0.1 nL, thereby decreasing reagent-associated costs while further increasing the throughput. However, this decrease in volume is also accompanied by an increase in the surface-to-volume-ratio, and therefore faces issues such as reagent absorption or evaporation. For these reasons, droplet microfluidics appears as a powerful alternative. Indeed, it enables us to carry out biochemical assays as well as cellular assays, with a high degree of control over a very low reaction volume (femtoliters to nanoliters). With the high frequencies of droplet generation (several hundred per second), droplet-based drug assays reach throughputs (up to millions of droplets screened in one experiment) and sensitivities comparable to well-based assays. Even though the majority of recent studies on high-throughput droplet-based microfluidics drug assays are based on fluorescence or absorbance readouts, other techniques such as Raman spectroscopy [76] or mass spectrometry [77,78] have also been successfully applied.

As a complementary measure, continuous flow microfluidics offers extremely rapid analysis (within seconds) at the cellular level [67]. In addition, this technique enables us to create interconnected tissues to study the impact of a single drug on several organs in newly-developed body-on-a-chip approaches [25]. Thus, microfluidics offers platforms for both extremely high-throughput screening of potential drugs and the possibility to test promising hits and their (side) effects in very short times. This promises faster and cheaper drug discovery compared to well plate-based drug assays in the long run.

## Figures and Tables

**Table 1:** Comparison of the three major technologies for drug assays.

Technique	Microtiter Well Plates	Droplet Microfluidics	Single Phase Microfluidics
Pros	Parallelizable	Extremely high parallel throughput	Very fast readout
	Miniaturization	Post processing possible	Continuous process possible
	Many commercial options available	Commercial options are currently emerging Highly customizable	Allows motility and viability assay High control over concentration via diffusion Highly customizable
Neutral	Current gold standard	Emerging technology (start-ups)	Niche technology
Cons	Long assay durations (days)	No industrial standard yet	Lower throughput
	Evaporation	Needs additional handling of oil and surfactants to generate droplets	Sample volumes are not isolated
	Local concentration gradients	Adding of compounds over time requires additional chip architecture	Unwanted diffusion Currently no commercial availability
